# Healthcare-related Infectious Diseases^1^

**DOI:** 10.3201/eid1011.040622_03

**Published:** 2004-11

**Authors:** Naomi Swanson, Clara Sue Ross, Kevin Fennelly

**Affiliations:** * Centers for Disease Control and Prevention, Atlanta, Georgia, USA;; † Environmental and Occupational Health Consultants, Inc., Cincinnati, Ohio, USA;; ‡ University of Medicine and Dentistry of New Jersey, Newark, New Jersey, USA

**Keywords:** healthcare, infectious diseases, women, conference summary

## Bloodborne Pathogens in the Workplace

Healthcare workers have approximately 600,000–800,000 exposures per year to HIV and hepatitis B and C. The U.S. Public Health Service published guidelines (June 2001) regarding prevention of exposure to bloodborne pathogens and recommendations for postexposure prophylaxis. Factors, such as nature of exposure or amount of body fluids, influence postexposure prophylaxis measures and can affect the effectiveness or use of prophylaxis. Several laws cover workers exposed to bloodborne pathogens, such as the Occupational Safety and Health Administration's Bloodborne Pathogen Standard, Workers' Compensation, and the Americans with Disabilities Act.

## Airborne Infections in Women at Work

A wide range of bacteria, viruses, and fungi can cause airborne infections in healthcare workers. The gender-based risk for diseases caused by many of these pathogens is not currently known. Female healthcare workers do appear to have the same risk for tuberculosis (TB) as men, although women may have a greater risk of progression to active TB. A number of airborne infections that healthcare workers can contract at the workplace can be prevented by vaccination (e.g., measles, influenza). Other types of infections (e.g., TB, severe acute respiratory syndrome) are best prevented by a combination of administrative, engineering, and personal protection controls.

